# Persistent Peri-Ablation Blood-Brain Barrier Opening After Laser Interstitial Thermal Therapy for Brain Tumors

**DOI:** 10.7759/cureus.37397

**Published:** 2023-04-10

**Authors:** Seamus Bartlett, Tavarekere N Nagaraja, Brent Griffith, Katelynn G Farmer, Meredith Van Harn, Sameah Haider, Rachel J Hunt, Glauber Cabral, Robert A Knight, O. Grahm Valadie, Stephen L Brown, James R Ewing, Ian Y Lee

**Affiliations:** 1 Neurosurgery, Wayne State University School of Medicine, Detroit, USA; 2 Neurosurgery, Henry Ford Health, Detroit, USA; 3 Radiology, Henry Ford Health, Detroit, USA; 4 Clinical Trials, Henry Ford Health, Detroit, USA; 5 Neurological Surgery, Henry Ford Health, Detroit, USA; 6 Neurology, Henry Ford Health, Detroit, USA; 7 Radiation Oncology, Henry Ford Health, Detroit, USA

**Keywords:** brain vascular permeability, tumor ablation, neuron specific enolase, ktrans, dce-mri

## Abstract

Purpose

Laser interstitial thermal therapy (LITT) is a minimally invasive, image-guided, cytoreductive procedure to treat recurrent glioblastoma. This study implemented dynamic contrast-enhanced magnetic resonance imaging (DCE-MRI) methods and employed a model selection paradigm to localize and quantify post-LITT blood-brain barrier (BBB) permeability in the ablation vicinity. Serum levels of neuron-specific enolase (NSE), a peripheral marker of increased BBB permeability, were measured.

Methods

Seventeen patients were enrolled in the study. Using an enzyme-linked immunosorbent assay, serum NSE was measured preoperatively, 24 hours postoperatively, and at two, eight, 12, and 16 weeks postoperatively, depending on postoperative adjuvant treatment. Of the 17 patients, four had longitudinal DCE-MRI data available, from which blood-to-brain forward volumetric transfer constant (K^trans^) data were assessed. Imaging was performed preoperatively, 24 hours postoperatively, and between two and eight weeks postoperatively.

Results

Serum NSE increased at 24 hours following ablation (p=0.04), peaked at two weeks, and returned to baseline by eight weeks postoperatively. K^trans^ was found to be elevated in the peri-ablation periphery 24 hours after the procedure. This increase persisted for two weeks.

Conclusion

Following the LITT procedure, serum NSE levels and peri-ablation K^trans^ estimated from DCE-MRI demonstrated increases during the first two weeks after ablation, suggesting transiently increased BBB permeability.

## Introduction

High-grade gliomas (HGGs) such as glioblastoma (GBM), a WHO Grade IV malignant astrocytoma, are among the most prevalent and aggressive primary brain tumors [1]. Despite significant advances in its molecular classification and an established standard of treatment, the median survival for GBM is only 12 to 15 months, with a two-year survival rate of 30% [2,3]. The standard of care is a combination of safe maximal surgical resection followed by radiotherapy and chemotherapy, primarily temozolomide [4]. However, the blood-brain barrier (BBB) prevents the entry of most drugs into the brain [5-7] and is a major obstacle in the effective treatment of GBMs [8]. Thus, even after treatment with radiotherapy and temozolomide, recurrence is nearly inevitable, and the median time for recurrence is 6.9 months [1,9]. Recurrence is often due largely to residual infiltrative glioma cells in the vicinity of the resection cavity, and notably, 90% of recurrent GBMs (rGBM) occur within a 2 cm margin of the primary tumor site [10-12].

Laser interstitial thermal therapy (LITT) is an emerging minimally invasive procedure used for its cytoreductive capabilities to treat rGBMs, as well as recurrent brain metastases, radiation necrosis, and epilepsy [13-15]. Thermal energy is absorbed from a laser fiberoptic catheter inserted stereotactically into the brain to ablate the tissue surrounding the laser emission tip through coagulative necrosis. The peri-ablation tissue is exposed to sublethal temperatures. The temperature distribution is monitored in real-time both spatially and temporally using magnetic resonance (MR) thermometry, thus allowing precise control of the ablation area, especially near functionally eloquent brain regions. LITT is also useful for deep-seated lesions that would be difficult to access via craniotomy, such as the insula and thalamus [16,17]. Of relevance, peri-ablation regions exposed to sublethal temperatures have been reported to demonstrate increased BBB permeability lasting from several days to weeks, in human patients and in murine glioma models [18,19].

To expand on these previous studies, this case series demonstrates a novel technique to analyze BBB permeability using a model selection paradigm that generates data-driven maps of model selection from DCE-MRI images to calculate the blood-to-brain forward volumetric transfer constant (K^_trans_^) for a contrast agent. These model maps provide a robust estimate of post-LITT vascular permeability. The analyses balance variance and bias in the estimation of model parameters and thus stabilize estimates of cerebral physiology produced by dynamic contrast-enhanced magnetic resonance imaging (DCE-MRI). Additionally, serum neuron-specific enolase (NSE) levels were measured, as NSE is a known peripheral marker of BBB disruption [20]. The main objective of this study was to determine the spatial and temporal characteristics of peri-ablation BBB disruption after LITT. Using a model selection analysis to estimate K^trans^ from unbiased DCE-MRI-derived model maps [21-23] and by measuring serum NSE, temporal changes in post-LITT BBB permeability were evaluated following tumor ablation.

## Materials and methods

Patient Selection

This study was conducted at Henry Ford Hospital, Detroit. Seventeen adult patients (eight females and nine males; mean age at treatment = 47.7±16.8 years) with radiological evidence of recurrent HGG (WHO grade 3 or 4) were screened for eligibility to participate (IRB 10934). Written informed consent was obtained from each participant prior to any study-related activity. Patients with a prior diagnosis of low-grade glioma (grade 2) were eligible if the recurrent tumor had radiographic characteristics of HGG, primarily the presence of contrast enhancement. Recruited patients also had lesions that were suitable for LITT if they met the following criteria: 1) supratentorial, 2) unilateral, 3) relatively well-circumscribed, 4) the lesion could be ablated by two 2-3 cm cylinders, i.e., two catheter trajectories, 5) the lesion was approached safely, and 6) the patient’s body habitus allowed for treatment in the intraoperative MRI. A biopsy of the lesion was obtained immediately prior to LITT through the same trajectory, and patients were eligible for this study if the biopsy showed recurrent HGG. Even if the biopsy did not show HGG and instead demonstrated treatment effect, the patients still underwent LITT, as LITT is an efficacious adjuvant therapy; those patients were not included in the current study.

Study Design

The objective of this study was to evaluate signs of BBB disruption after LITT using NSE and DCE-MRI biomarkers as signatures of that disruption. Patients underwent pre-LITT baseline DCE-MRI and NSE measurements within 24 hours prior to LITT. As an addition to the routine tumor volume protocol, a post-LITT DCE-MRI was performed within 24 hours after LITT and between two and eight weeks post-LITT. Serum NSE was also measured at these time points. LITT was performed as previously described [15].

DCE-MRI protocol

Standard tumor volume protocol with added DCE-MRI was obtained within 24 hours prior to and within 24 hours after LITT, then between two and eight weeks after LITT. All patients were scanned using a 3 Tesla Philips Insignia MRI scanner to eliminate machine-to-machine variability. Intraoperative MRI scans were obtained on a 1.5 Tesla Philips Achieva MRI scanner as typically required for the LITT procedure.

The tumor volume protocol consisted of pre-contrast T1-weighted, T2-weighted, fluid-attenuated inversion recovery (FLAIR), arterial spin labeling (ASL, for blood flow), and six-direction diffusion tensor imaging (DTI) studies, followed by a variable tip-angle (six tip-angles) SPGR data set for pre-contrast T1 mapping. The DCE-MRI sequence was started, and after the acquisition of 30 seconds of data, a split dose of Magnevist was administered and followed for six minutes. Following this, the second half-dose of Magnevist was administered in an echo-planar dynamic susceptibility (DSC) study for perfusion estimation and tumor blood volume estimation. A post-contrast T1-weighted series of images completed the imaging protocol. The MRI data were processed offline to produce quantitative parameter maps following published procedures [20-22]. Values for the DCE-MRI parameters were estimated for the pre-LITT and post-LITT sessions. The post-LITT maps and T1 ‘post minus pre’ subtraction as well as cerebral blood flow images were used to verify the location and extent of the tumor.

Measurement of Serum Biomarker Levels of BBB Disruption

Patients had their blood drawn for serum NSE measurements preoperatively and postoperatively at 24 hours, two weeks, eight weeks, 12 weeks, and 16 weeks, depending on the adjuvant treatment received postoperatively. Serum NSE was measured using human neuron-specific enolase ELISA kits (MyBioSource, Inc., San Diego, CA) following the manufacturer’s instructions.

Statistical Analysis

Parametric maps for vascular permeability from DCE-MRI were segmented by model selection into pixels with increased K^trans^ values that suggested elevated BBB permeability. NSE data were normalized with the pre-LITT value set at one and analyzed using nonparametric sign tests (equivalent to the paired t-test) to compare normalized NSE data at the various time points. The level of statistical significance was set at p<0.05. All analyses were performed using SAS 9.4 (SAS Institute Inc., Cary, NC).

## Results

Twelve of the 17 patients underwent ablation with the Medtronic Visualase LITT system (Medtronic, Inc., Minneapolis, MN) and five with the Monteris Neuroblate system (Monteris, Plymouth, MN) between May 2017 and December 2019. All surgeries were performed by a single surgeon, and all patients previously underwent treatment with external beam radiation and temozolomide chemotherapy. Pathology at the time of ablation demonstrated five patients with GBMs: five with anaplastic oligodendrogliomas, two with anaplastic astrocytomas, one with anaplastic ependymoma, one with spindle cell sarcoma, one with radiation necrosis, and one with a residual glioma with gliosis. One patient had no neoplastic tissue on biopsy but a history of pleomorphic xanthoastrocytoma. The median extent of ablation was 95.9+7.3%, of which 12 patients had gross total ablation. Patient characteristics are summarized in Table 1.

**Table 1:** Summary of patient characteristics PtN: patient number; EoA: extent of ablation; TMZ: temozolomide; RT: radiation therapy; GBM: glioblastoma multiforme

PtN	Age	Histology at LITT	Biomarkers	Initial diagnosis	Location	EoA	TMZ/RT	System
1	58	Spindle cell sarcoma	TERT mutant	Anaplastic oligodendroglioma	Right temporal	85	Yes	Visualase
2	81	Recurrent GBM	IDH1 wild-type, 1p, 19q intact; Methylated MGMT	GBM	Right frontal	100	Yes	Visualase
3	41	Radiation necrosis (treatment effect)	IDH1 R132H, 1p, 19q LOH Methylated MGMT	GBM	Left frontal	90	Yes	Visualase
4	55	Recurrent GBM	IDH1 wild-type, Methylated MGMT	GBM	Left temporal	100	Yes	Visualase
5	29	Recurrent oligodendroglioma	IDH mutant Oligo features 1p19q LOH, Methylated MGMT	Oligodendroglioma	Left parietal	100	Yes	Visualase
6	30	Recurrent anaplastic ependymoma	MEN1, smarcB1, ATRX, CDKN2A/2B loss	Anaplastic ependymoma	Right frontal	95	Yes	Visualase
7	37	Residual glioma with gliosis	IDH1 R132H, p53	Anaplastic astrocytoma	Left temporal	100	Yes	Visualase
8	37	Anaplastic astrocytoma	IDH1 R132H, 1p, 19q intact, Unmethylated MGMT	Oligodendroglioma	Right frontal	100	Yes	Visualase
9	69	Recurrent astrocytoma	EGFR viii, IDH1 wild-type	Diffuse fibrillary astrocytoma	Right frontal	100	Yes	Visualase
10	57	GBM	IDH1 wild-type, Unmethylated MGMT	GBM	Right parietal	100	Yes	Visualase
11	25	GBM	IDH1 wild-type, NF1, ATRX, CDKN2A/B loss	GBM	Left midbrain	80	Yes	NeuroBlate
12	26	No neoplastic tissue	BRAF V600e mutation present	Pleomorphic xanthoastrocytoma	Right parietal	100	Yes	NeuroBlate
13	36	Recurrent anaplastic oligodendroglioma	IDH1 R132H, TP53	Oligodendroglioma	Left insular	80	Yes	NeuroBlate
14	56	Recurrent anaplastic oligodendroglioma	IDH1 R132H, 1p, 19q co-deleted	Oligoastrocytoma	Right insular	100	Yes	NeuroBlate
15	39	Recurrent anaplastic oligodendroglioma	IDH1 R132H, 1p, 19q co-deleted	Anaplastic oligodendroglioma	Bilateral frontal	100	Yes	Visualase
16	62	Recurrent anaplastic oligodendroglioma	IDH1 R132H, 1p, 19q co-deleted, Unmethylated MGMT	Anaplastic oligodendroglioma	Left frontal	100	Yes	Visualase
17	65	Recurrent GBM	IDH1 R132H, 1p, 19q co-deleted, Unmethylated MGMT	GBM	Right frontal	100	Yes	NeuroBlate

The average length of hospital stay was three days, with a range of one to 22 days. One patient with a hospital stay of 22 days experienced a deep vein thrombosis that caused a prolonged intensive care unit (ICU) stay of greater than 96 hours and was later diagnosed with heparin-induced thrombocytopenia. Other complications included one patient with a urinary tract infection (UTI), two patients with postoperative urinary retention, and three patients who experienced a new neurological deficit. Neurological deficits included a patient with new numbness involving the left side of the body, particularly in the left upper extremity extending to the torso up to the face; another with worsening mental status along with left-sided neglect; and one with significant postoperative alteration of consciousness. Two patients returned to the operating room (OR) within 90 days following laser ablation. One patient underwent right frontoparietal awake craniotomy 10 weeks following ablation for tissue diagnosis, relief of mass effect, and cytoreduction after MRI demonstrated worsening enhancing lesions posterior to the ablation zone (Appendix, Figure 4). Another patient had a ventriculoperitoneal shunt replaced 10 weeks after ablation for progressive enlargement of the left lateral ventricle. One patient was readmitted within 30 days for stroke-like symptoms, including progressive weakness and verbal difficulty, although computed tomography (CT) of the head and CT angiography showed no imaging findings to suggest a stroke.

Serum NSE

**Figure 1:**
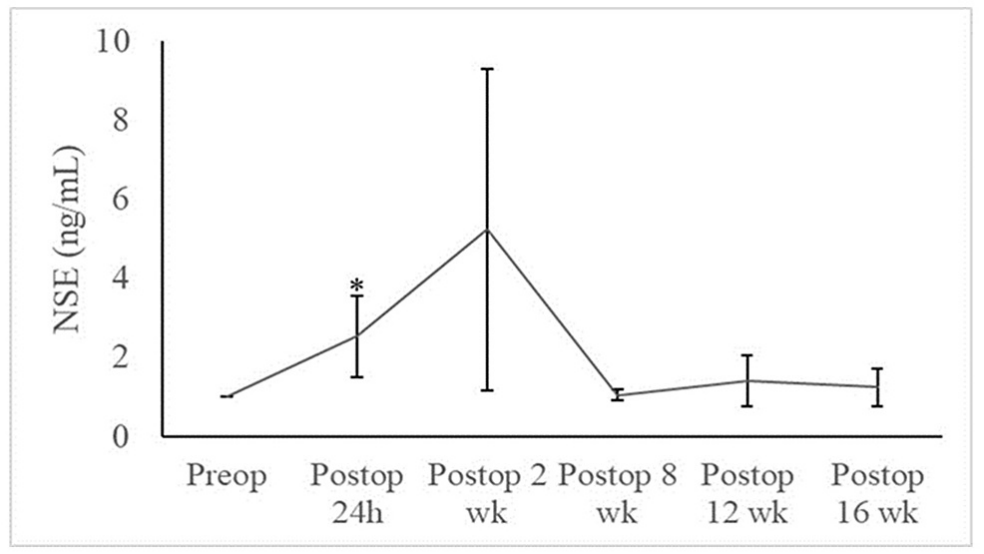
Normalized serum NSE levels over time There was a clinically significant increase in the mean serum NSE level at 24 hours postoperatively (*p=0.049) compared to pre-operative levels. An increase in NSE, albeit with high variability, persisted at two weeks postoperatively. No other changes were statistically significant.

Compared to preoperative levels, normalized NSE values increased 24 hours after ablation and peaked two weeks after the procedure (Figure 1). Subsequently, serum NSE decreased eight weeks postoperatively and continued to remain around that level at 12 weeks and 16 weeks postoperatively. The increase in the mean serum NSE level at 24 hours postoperatively was statistically significant (p=0.04) compared to preoperative levels. No other comparisons were found to be statistically significant.

DCE-MRI

**Figure 2:**
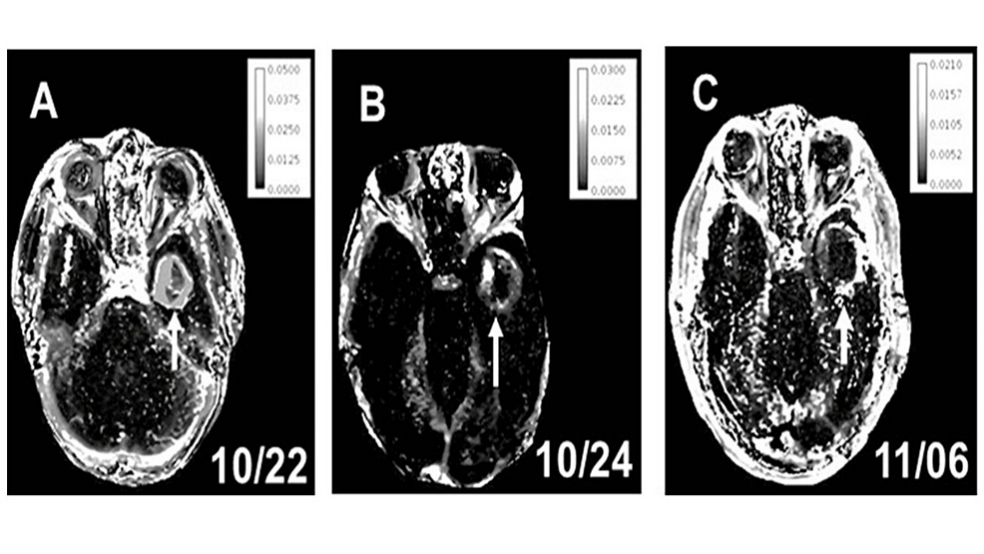
A set of three Ktrans maps from one LITT case Before LITT, the leaky vasculature is characterized by elevated K^trans^ values limited to the tumor core (A, arrow). At 24 hours post-LITT (B, arrow), the peri-ablation periphery exhibits increased K^trans^ values that persisted until two weeks post-LITT (C, arrow). The scale bars within each map give K^trans^ values from the lowest to the highest (dark gray to white). Note the bright, intra-tumoral pixels shifting to the peri-ablation periphery after LITT.

Of the 17 patients, DCE-MRI-generated K^trans^ maps could be constructed for four patients with longitudinal monitoring. The patients are listed in numbers one through four in Table 1. All four patients had a history of HGG and a similar extent of ablation. Intra-tumoral K^trans^ was found to be elevated prior to laser ablation, with peri-ablation elevations persisting after the procedure for two weeks (Figure 2).

**Figure 3:**
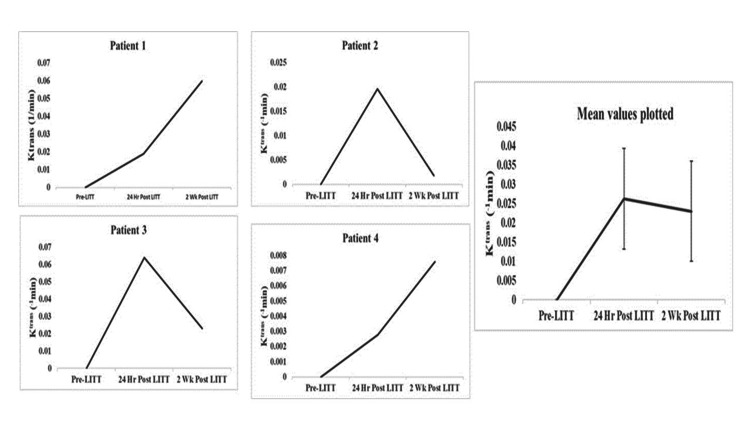
Peri-ablation Ktrans values for each of the four patients with DCE-MRI-generated Ktrans maps plotted over time The average K^trans ^values are shown in the bottom right graph. Based on the model selection paradigm, the pre-LITT peri-tumoral regions in all patients showed only plasma volume (v_p_) values with no evidence of leakage, i.e., K^trans^=0. After ablation, these regions were segmented as models two or three with increased peri-ablation K^trans^ values. They displayed an increase in ^Ktrans ^values from pre-LITT to 24 hours post-LITT in all cases. Overall, there was a gradual increase in K^trans^ values from pre-LITT to two weeks following the procedure.

**Figure 4:**
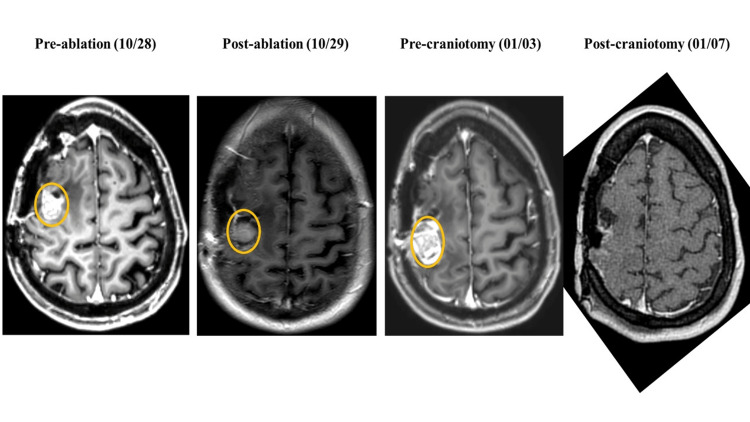
Images from one case with tumor ablation followed by craniotomy to relieve mass effect and for tissue diagnosis and cytoreduction. This patient underwent right frontoparietal awake craniotomy about 10 weeks following ablation for tissue diagnosis, relief of mass effect, and cytoreduction. This procedure was necessary after MRI demonstrated worsening, enhancing lesions posterior to the ablation zone. The yellow ovals in the first three panels indicate the brain regions that were treated first by LITT and then by craniotomy-based cytoreduction. The nearly complete removal of the enhancing mass is visible in the fourth panel.

All patients underwent pre-procedure DCE-MRI, with a mean tumoral K^trans^ of 0.0351+0.024 (min-1). Following LITT, K^trans^ maps shifted away from the tumor body to the peri-ablation zone at 24 h with a mean value of 0.0263+0.026 and remained elevated in that zone at two weeks with a mean value of 0.0230+0.032. The mean peri-ablation K^trans^ values over time for each of the four patients with DCE-MRI-generated K^trans^ maps, as well as their mean, are shown in Figure 3. While processing the images for K^trans^, the index of vascular volume, v_p_, was also computed. Changes in v_p_ values reflected the status of the vasculature; the mean pre-ablation values which were 0.013±0.004% decreased to 0.009±0.007 at 24 hours and remained at 0.009±0.002 at two weeks post-LITT.

## Discussion

Herein, we report findings on the localization and quantification of increased peri-ablation BBB permeability following LITT for recurrent HGG. These studies demonstrated a ring of increased contrast enhancement in the periphery of the ablation zone (i.e., the peri-ablation zone) on post-contrast T1-weighted images that persisted for weeks after tumor ablation. Confirming a previous clinical report [18], elevated peri-ablation K^trans^ values with concurrently increased serum NSE levels were also observed. The v_p_ values with concurrently increased serum NSE levels were also observed and decreased overall, probably due to decreased tumor-associated hyperplasic vasculature after ablation. A summary of the histopathological findings of all recruited patients is shown in Table 1. However, please note that the MRI data were acquired from the first four patients in this table. All of them had a history of high-grade gliomas, and they all had a similar extent of ablation treated with Visualase, ranging from 85%-100%. Of these four, serum NSE increased, as did K^trans^ at 24 hours post-ablation. For one patient (patient #2 in the table) with radiation necrosis on biopsy at the time of LITT, serum NSE decreased at 24 hours post-ablation but increased at two weeks. However, K^trans^ values for this patient were found to be elevated at both 24 hours and two weeks. Broadly, a linear relationship was observed between elevated K^trans^ and serum NSE levels in patients with GBM.

LITT is a minimally invasive cytoreductive procedure used to ablate tumors that offers an alternative to open resection. BBB disruption associated with laser ablation, both in human and animal models, has been reported. Previous mouse models have used histopathology and immunofluorescence to show leakage of both small and large molecules, including human IgG (~150 kDa) and doxorubicin, across the BBB following LITT [19]. The permeability of small and large molecules was a result of the disruption of endothelial tight junctions. An increase in endothelial cell transcytosis was also detected following LITT on electron microscopy, which could aid in BBB permeability. Median survival was assessed following doxorubicin injection and increased by 71% in the LITT plus doxorubicin group compared to the historical control group, illustrating how LITT yields the potential for future investigations into the possibility of bypassing the BBB to administer adjuvant chemotherapy [19]. A recent study employing MRI-guided LITT in an animal glioma model also reported an acute increase in peri-ablation BBB permeability [23].

In addition to animal models, BBB permeability has been examined in human subjects as well. Leuthardt et al. noted a temporary opening in the BBB that peaked at one to two weeks and resolved within four to six weeks. K^trans^, an MRI measure of the rate constant at which vascular fluids pass into the interstitium, and serum NSE levels, a peripheral marker of increased BBB permeability, were examined as measures of BBB disruption. It was reported that K^trans^ elevated promptly following laser ablation and declined over four weeks. NSE was found to be elevated following ablation, reaching its maximum within one to three weeks, and resolving within six weeks [18].

The present case series measured serum NSE and utilized a data-driven technique to generate maps of model selection and robust estimates of vascular parameters from DCE-MRI data. DCE-MRI observed an increase in contrast enhancement in the periphery of the ablation zone and thus assessed leakage across the BBB both quantitatively and qualitatively. Using the temporal variation in six minutes of T1 maps, model maps were generated, allowing for robust estimates of K^trans^ and describing leakage of the BBB. This technique performs a data-driven voxel-by-voxel segmentation of the brain into three regions, labeled model 1, model 2, and model 3, according to the number of model parameters that can be estimated. This segmentation is based on a physiologically nested description of the vasculature in each voxel. It aims to balance bias and variance in estimating vascular properties and thus generate robust estimates of model parameters. Model 1 regions correspond to a normal brain and an intact blood-brain barrier; they display only vascular volume (v_p_) with microvascular leakage but not K^trans^ and its consequence, interstitial volume fraction (v_e_) for the contrast agent because they cannot be stably estimated. Model 2 regions, corresponding to regions with weakly leaking microvessels, include voxels in which extravasation of contrast agent (K^trans^) and its extravascular distribution (v_e_) are measurable, but no evidence of backflux (k_ep_) from the interstitial compartment is present in the time trace of T1. Finally, model 3 regions, corresponding to regions with markedly high BBB breakdown, highlight regions with v_p_, K^trans^, as well as nonzero k_ep_ [20-22]. Thus, in comparison to model 1 regions, voxels assigned to models 2 or 3 show definite signs of vascular leakage. The model selection paradigm segments the brain into regions with no BBB leakage, with only blood-to-brain transfer, or with both blood-to-brain and backflux. Of these, regions with blood-to-brain transfer and those with backflux, both exhibit increased K^trans, ^whereas only regions with backflux show measurable v_e_ values. Therefore, K^trans^ was chosen as the primary index of increased vascular permeability.

This data-driven approach, by imposing a constraint on the number of parameters to be estimated, stabilizes estimates of the available parameters and minimizes bias in those estimates. In contrast, Leuthard et al. employed a manual selection technique to isolate a peri-ablation region with elevated K^trans^ that was superimposed onto the maps from later imaging sessions to derive subsequent K^trans^ values [18].

BBB breakdown, signaled by model 2 and 3 regions and non-zero K^trans^, was seen to be present for two weeks post-LITT in the present study; there was a corresponding rise in serum NSE. This signals a temporary disruption of the BBB for two weeks following laser ablation, which returned to baseline by eight weeks postoperatively. An opportunity to bypass the BBB in the locale of the treated lesion might be provided by this transient BBB leakage and should be further explored as a mechanism for administering adjuvant chemotherapy. Supportive of this suggestion, a phase II clinical study reported increased overall survival after LITT followed by six weekly treatments with doxorubicin compared to historic controls [24].

Increased BBB permeability to small molecules is reported to be sustained for several weeks after acute localized injuries such as cerebral ischemia [25]. Therefore, returning to baseline levels of serum NSE (78 kDa) may not exclude the continued passage of smaller molecules across the transiently compromised BBB. Additional investigations should be done to delineate the size-selective qualities of the BBB permeability, which could aid in drug delivery.

One limitation of this study was that patient 12 showed no neoplastic tissue on biopsy at the time of LITT. This could be attributed to a sampling error as the lesion was small on imaging and the patient has a history of HGG. Another limitation of this study is the small sample size of the DCE-MRI-generated K^trans^ maps, which could only be generated for four patients. This is partly due to the technical and logistical difficulties associated with the new DCE-MRI model selection analysis that was applied to clinical data. The protocol is challenging as it requires four points to construct a trend. Additionally, DCE-MRI demands lengthy scans. Any patient movement during imaging will render the data unusable. A combination of not obtaining any valuable data from the scans as well as performing routine clinical scans in many cases contributed to the small sample size. Furthermore, unfortunately, DCE-MRI was not measured at a consistent time interval between the patients as a result of the adjuvant postoperative treatment. Due to the small sample size, this study needs to be replicated on a larger scale with a more consistent DCE-MRI follow-up.

## Conclusions

In summary, this case series demonstrates a novel method to detect transiently altered peri-ablation zone BBB permeability, both spatially and temporally, following MRI-guided LITT with DCE-MRI model selection analysis, correlated with serum NSE level measurement. Both DCE-MRI and serum NSE levels exhibited increased peri-ablation BBB permeability for two weeks; the increased permeability returned to baseline by eight weeks post-LITT. These observations provide independent confirmation of previous reports. The increase in BBB permeability following LITT should be investigated further as a potential approach to overcome the challenge that the BBB poses when delivering adjuvant chemotherapy. Additional research should be conducted to define the size-selective characteristics of the BBB disruption to aid in drug delivery.
